# Development of a Community-Based e-Health Program for Older Adults With Chronic Diseases: Pilot Pre-Post Study

**DOI:** 10.2196/33118

**Published:** 2022-01-17

**Authors:** Vivien Xi Wu, Yanhong Dong, Poh Choo Tan, Peiying Gan, Di Zhang, Yuchen Chi, Felicia Fang Ting Chao, Jinhua Lu, Boon Heng Dennis Teo, Yue Qian Tan

**Affiliations:** 1 Alice Lee Centre for Nursing Studies Yong Loo Lin School of Medicine National University of Singapore Singapore Singapore; 2 Healthy Longevity Translational Research Programme Yong Loo Lin School of Medicine National University of Singapore Singapore Singapore; 3 Department of Medicine Yong Loo Lin School of Medicine National University of Singapore Singapore Singapore; 4 Changi General Hospital Singapore Singapore; 5 Sengkang General Hospital Singapore Singapore; 6 Department of Microbiology and Immunology Yong Loo Lin School of Medicine National University of Singapore Singapore Singapore; 7 Immunology Translational Research Programme Yong Loo Lin School of Medicine National University of Singapore Singapore Singapore

**Keywords:** eHealth, self-management, older adults, chronic disease, community care, elderly, community, innovation, development, pilot, evaluation, health literacy, empowerment, feasibility, engagement

## Abstract

**Background:**

Chronic diseases may impact older adults’ health outcomes, health care costs, and quality of life. Self-management is expected to encourage individuals to make autonomous decisions, adhere to treatment plans, deal with emotional and social consequences, and provide choices for healthy lifestyle. New eHealth solutions significantly increase the health literacy and empower patients in self-management of chronic conditions.

**Objective:**

This study aims to develop a Community-Based e-Health Program (CeHP) for older adults with chronic diseases and conduct a pilot evaluation.

**Methods:**

A pilot study with a 2-group pre- and posttest repeated measures design was adopted. Community-dwelling older adults with chronic diseases were recruited from senior activity centers in Singapore. A systematic 3-step process of developing CeHP was coupled with a smart-device application. The development of the CeHP intervention consists of theoretical framework, client-centric participatory action research process, content validity assessment, and pilot testing. Self-reported survey questionnaires and health outcomes were measured before and after the CeHP. The instruments used were the Self-care of Chronic Illness Inventory (SCCII), Healthy Aging Instrument (HAI), Short-Form Health Literacy Scale, 12 Items (HLS-SF 12), Patient Empowerment Scale (PES), and Social Support Questionnaire, 6 items. The following health outcomes were measured: Montreal Cognitive Assessment, Symbol Digit Modalities Test, total cholesterol (TC), high-density lipoproteins, low-density lipoproteins/very-low-density lipoproteins (LDL/VLDL), fasting glucose, glycated hemoglobin (HbA_1c_), and BMI.

**Results:**

The CeHP consists of health education, monitoring, and an advisory system for older adults to manage their chronic conditions. It is an 8-week intensive program, including face-to-face and eHealth (*Care4Senior* App) sessions. *Care4Senior* App covers health education topics focusing on the management of hypertension, hyperlipidemia, and diabetes, brain health, healthy diet, lifestyle modification, medication adherence, exercise, and mindfulness practice. Content validity assessment indicated that the content of the CeHP is valid, with a content validity index (CVI) ranging 0.86-1 and a scale-CVI of 1. Eight participants in the CeHP group and 4 in the control group completed both baseline and post intervention assessments. Participants in the CeHP group showed improvements in fasting glucose, HbA_1c_, TC, LDL/VLDL, BMI, SCCII indices (Maintenance, Monitoring, and Management), HAI, and PES scores post intervention, although these changes were not significant. For the participants in the control group, the scores for SCCII (management and confidence) and HLS-SF 12 decreased post intervention.

**Conclusions:**

The CeHP is feasible, and it engages and empowers community-dwelling older adults to manage their chronic conditions. The rigorous process of program development and pilot evaluation provided valid evidence to expand the CeHP to a larger-scale implementation to encourage self-management, reduce debilitating complications of poorly controlled chronic diseases, promote healthy longevity and social support, and reduce health care costs.

## Introduction

The life expectancy of Singaporeans has increased from 76.1 years in 1990 to 84.8 years in 2017 [1]. Multiple socioeconomic factors impact the quality of life among older individuals, including functionally limiting diseases [2]. It is estimated that Singaporeans spend the last 10.6 years in poor health conditions owing to a higher prevalence of chronic diseases [1]. These commonly include diabetes, hypertension, and lipid disorders, which may severely impair the quality of life [3]. Not only the quantity of time spent alive but also quality of life are important. For community-dwelling older adults with chronic diseases, self-management is essential to their quality of life [4]. They are more susceptible to further cognitive impairment, which could drastically compromise their ability of performing self-care [5,6]. Hence, self-management programs allowing early detection and prevention of cognitive impairment can substantially improve healthy aging [7].

Self-management refers to daily activities that individuals take for themselves and families to stay healthy and to care for long-term illness [8]. Individuals are more involved in their self-care and given the opportunity to adopt an active program suitable for their medical conditions [9]. Effective self-management helps older adults to experience improved health outcomes and to reduce medical cost [10].

Self-management is expected to make the health care system more patient-centric by shifting responsibilities toward individuals in terms of making autonomous decisions, adhering to treatment plans, and dealing with emotional and social consequences caused by their medical conditions [9]. It also provides choices for individuals to live in an active and healthy lifestyle. Self-management requires older adults to have a good understanding of the disease itself, the prescribed medication, as well as other procedures related to self-care [11]. Health literacy is the capacity of individuals to obtain, process, and understand basic health information and the services needed to make appropriate health decisions [12]. Hence, improvement in health literacy of chronic disease management empowers older adults to have access, understand, and use health information to make decisions when performing self-management [13]. According to the SIGNS Study conducted by the Centre for Ageing Research and Education, health illiteracy is prevalent among the older Singaporeans [14]. With improved health literacy, community-dwelling older adults are expected to improve their self-management skills [13], helping them to make decisions on suitable health practice and strategies to cope with their chronic diseases.

Additionally, social support from family, friends, and neighbors serves as a complementary strategy for enhancing an individual’s self-management skills [15] by providing relevant information, emotional support, and practical help [16]. Social support is closely related to health behavior and health outcomes. For people who have lower economic status or educational levels and who are more socially isolated, educational and counseling interventions developed for their self-management of chronic diseases may be less effective [17]. In this context, social support can significantly compensate for their inequality in health [15]. Daviglus et al [18] developed the concept that family and social network supports play an essential role in keeping the chronic disease under control. Hence, social support significantly contributes to the adherence to the treatment plan as well as self-management behavior [19].

The modern health care system leverages on innovation and technologies to empower patients and families in self-care. Compared to conventional approaches to patient education, new eHealth solutions such as mobile health, web-based learning, and telehealth significantly increase patients’ health literacy and empower them in self-management [20-22]. Multiple studies have shown that eHealth solutions have positive impacts on chronic disease management through effective patient education and increased medication adherence [23,24]. Therefore, eHealth is actively promoted powered by information technology. Health illiteracy among the older population has negative health outcomes and increases health care costs [14]. It is a critical issue for a rapidly aging population in a technology-driven society.

In an aging population, it is necessary to shift the health care landscape toward the community to ease the burden of acute hospitals [25,26]. Such community health care support is provided by multidisciplinary primary care teams to promote effective self-management [27,28]. Having health professionals constantly motivate and support patients to manage the chronic conditions signifies a supportive relationship, thus promoting effective self-management for patients to pursue [28]. In light of the aforementioned gaps and the potential use of technology in bridging these needs, this study aims to develop a Community-Based e-Health program (CeHP) for older adults with chronic diseases and to conduct a pilot evaluation prior to a full-scale interventional program.

## Methods

### Research Design

This study describes a systematic 3-step process of developing the CeHP, coupled with the use of a smart device application. A pilot study with a 2-group pre- and posttest repeated measures design was adopted.

### Setting

Subjects were recruited from Community Nurse Posts at two senior activity centers (SACs) within the neighborhood in the east region of Singapore. The strategic location ensures that the nursing service is convenient and accessible for the older adults living in the community. The services consist of health screening, individual and group health coaching, health and geriatric assessment, chronic disease monitoring and education, care referral and coordination, and complex nursing care [29].

### Developing the Intervention

Development of the CeHP consisted of a systematic 3-step process: theoretical framework, a client-centric participatory action research process, content validity assessment and pilot testing.

#### Theoretical Framework

A systemic scoping review identified the focus of the theoretical approaches was behavior change in most of the self-management programs (SMPs). The most frequently used theory was the social cognitive theory, where the participants’ self-efficacy increased as a result of the SMPs, and evidence showed that the associated behavior change could affect various health outcomes [30]. Building on the theoretical foundation, we adopted the concept of self-management, which can capture the complexity of living with medical conditions and managing it in an individual’s everyday life [31]. A diverse body of knowledge reflects a number of factors that influence the individual’s self-management. The factors influencing self-management are categorized into (1) health status: medical condition; (2) individual: age, gender, self-efficacy, integration, diversity; (3) family: socioeconomic status, family function; and (4) environmental: social networks, community, and health care system [32].

**Figure 1:**
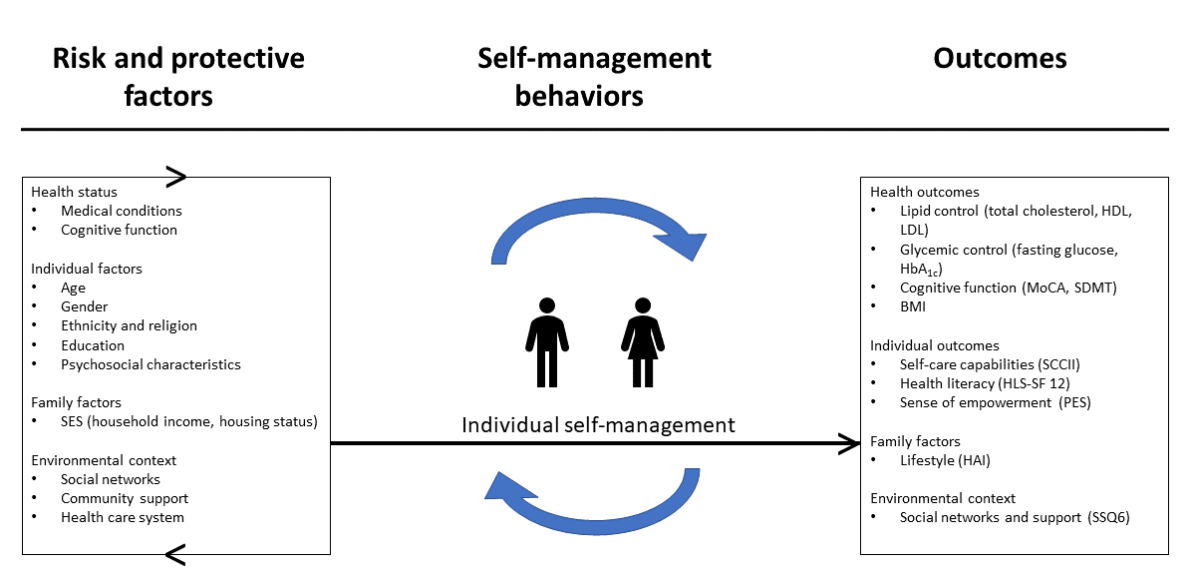
Self-management framework. HAI: Healthy Aging Instrument, HbA_1c_: glycated hemoglobin, HDL: high-density lipoprotein, HLS-SF 12: Short-Form Health Literacy Scale, 12 Items, LDL: low-density lipoprotein, MoCA: Montreal cognitive assessment, PES: Patient Empowerment Scale, SCCII: Self-care of Chronic Illness Inventory, SDMT: symbol digit modalities test, SES: socioeconomic status, SSQ6: Social Support Questionnaire, 6 items. [32].

The individual’s self-management is interactive and influences a variety of health outcomes, especially for individuals living with chronic conditions. In our study, the following outcomes in relation to the factors are measured: (1) health outcomes: cognitive function (Montreal Cognitive Assessment [MoCA], Symbol Digit Modalities Test [SDMT]), lipid profile (total cholesterol [TC], high-density lipoprotein [HDL] cholesterol, and low-density lipoprotein [LDL]/very-low-density lipoprotein [VLDL] cholesterol), glycemic profile (fasting glucose and glycated hemoglobin [HbA_1c_]), and BMI; (2) individual outcomes: sociodemographics, self-care capabilities (Self-care of Chronic Illness Inventory [SCCII]), health literacy (Short-Form Health Literacy Scale, 12 Items [HLS-SF12]), empowerment (Patient Empowerment scale [PES]); (3) family outcomes: lifestyle (Healthy Aging Instrument [HAI]); and (4) environmental outcomes: social networks and support (Social Support Questionnaire, 6 items [SSQ6]) (Figure 1) [32]. Self-management may influence how environmental resources, such as health care system and community support, are accessed and utilized, and the nature of interactions with health care professionals. Reasonable targets could be set with the potential to alter the behavioral changes and health outcomes. For example, interventions may target at psychosocial factors, family functioning, or working with the individuals to develop and enhance the self-management capabilities. Hence, CeHP is targeted at working with the individuals to develop and enhance their self-management capabilities.

#### Client-Centric Participatory Action Research Process

CeHP was developed to promote older adults’ self-management capabilities with their chronic conditions. CeHP was designed through a 3-stage iterative, client-centric, participatory action research process [33]. First, a front-end analysis was conducted to identify the unique health care needs of older adults and initial design ideas through focus groups and literature search. Second, a preliminary design of the intervention was developed from the literature and focus group findings. Finally, we iteratively incorporated revisions and refinements on the basis of client-centric feedback, which was collected during usability sessions.

##### Stage 1: Front-End Analysis

A comprehensive search and evaluation of existing eHealth interventions were carried out. Evaluation from the evidence-based literatures provided a fundamental understanding of the current interventions. A systematic review examined community-based SMPs for older adults with chronic conditions and evidenced that SMPs involved fostering skills to improve problem-solving, health behavior, and disease management [34]. However, this review highlighted that SMPs need to broaden the strategies to be more patient-centered by helping older adults manage the impact of the conditions on their daily lives, and to provide strategies for managing interacting symptoms, treatments, and everyday problems due to the high prevalence of multiple morbidities in older adults [34]. A recent meta-analysis provides evidence that educational intervention is effective to improve the health literacy of the perceived severity and susceptibility of the patients’ medical conditions, and perceived benefits of the treatment, and increased medication adherence among adult patients diagnosed with hypertension, hyperlipidemia, or diabetes [35]. The educational interventions consist of face-to-face counseling with the participants on the diseases, complications, medications, side effects, adherence, lifestyle changes, self-monitoring, and self-management skills [35].

We have conducted a meta-analysis on the technology-based interventions on diabetes, and the results indicate that technology-based psychosocial interventions had significant effects on diabetes distress, self-efficacy, and HbA_1c_ levels in adults with type 2 diabetes mellitus (T2DM) [36]. The technology-based interventions consist of telephone-based health coaching, telemonitoring, computer-assisted self-management, web-based programs and tools, and mobile apps. Nevertheless, an integration of technology-based psychosocial interventions into usual care can enhance treatment for older adults with T2DM [36]. In the current COVID-19 pandemic situation, telehealth applications have provided solutions to facilitate the care of older adults with chronic conditions, and the medical apps delivered virtual assessments and treatments, improved medication adherence for older adults, and supported the health care professionals during the pandemic [37].

Our researchers conducted focus groups with older adults to explore their needs regarding eHealth. Three focus group discussions were conducted, and the thematic analysis was carried out. Three major themes emerged from the analysis: (1) personal approach in living with chronic diseases (older adults applied positive thinking and accepted the needs to change their habits and follow the instructions of health care professionals); (2) navigating health-related information (older adults obtained health information from health care professionals, health talks from reputable organizations, experiences of friends or family, internet resources, talk shows in the media, and web-based videos); and (3) decision-making on sieving credible eHealth information (older adults often experienced online health resources are overwhelming and confusing, they either turn to health care professionals for advice or use own experience and knowledge to judge the reliability and credibility of the web-based health resources). Details of the qualitative study will be published in a subsequent paper. In addition, our recent scoping review also highlighted the concerns of older adults on the barriers of web-based interventions, such as the lack of access and proficiency in technology, or the lack of interest in the use of digital technologies [37].

##### Stage 2: Design and Development

With inputs from the literature and focus groups, the researchers developed the preliminary contents of the CeHP. Based on client-centric design suggestions, the following principles guided the development of the CeHP: (1) the intervention must be designed for older adults, (2) the content must be related to the specific health knowledge deficits that were identified during focus group and literature evaluations, and (3) the content needs to be delivered in a brief and skimmable format to fit the attention span and cognitive capabilities of the older adults. The details of the contents are presented in the Results section.

##### Stage 3: Formative, User-Centric Evaluation

Formative evaluation took the form of multimodal usability testing [38,39] which sought to elicit feedback on applicability, content, ease of use, acceptance, and time to complete the modules. Feedback was collected from the participants during the development of the intervention on the usage information and usability testing, which were subsequently used to further extend and refine the intervention [40].The formative evaluation generated input regarding revisions and modifications that informed the design and development of the CeHP.

#### Content Validity Assessment

A committee of experts was formed to evaluate the content validity of the CeHP, including 2 nurse clinicians, 2 physicians, a dietician, a physiotherapist, and a pharmacist, specifically on the clinical relevance and quality of the contents. They rated the contents from 1 (not relevant/appropriate/comprehensive) to 4 (very relevant/appropriate/comprehensive). Content experts were required to provide feedback if they had rated any learning point 2 and below on any of the aspects.

#### Pilot Test of the Study Intervention

##### Sampling and Recruitment Process

Convenience sampling was used. Recruitment was carried out through word of mouth and recruitment poster at 2 SACs. The inclusion criteria were as follows: (1) age ≥55 years; (2) being able to understand and communicate in either English or Chinese (Mandarin); (3) being able to give consent to participate; (4) living within the community setting; (5) being diagnosed with at least one of these chronic conditions (hypertension, hyperlipidemia, or diabetes mellitus); and (6) being able to commit to the 8-week CeHP. The exclusion criteria are as follows: (1) having severe cognitive impairment; (2) having severe psychiatric disorders; (3) having severe vision impairment; and (4) having severe hearing impairment. Participants in the intervention group were recruited from SAC 1, and they completed the CeHP regimen. Participants in the control group were recruited from SAC 2, and they continued with their usual recreational programs. Recruitment was carried out at 2 SACs at different physical locations to minimize contamination between the two groups.

### Data Collection Procedure

The questionnaires were administered at two time points: baseline and post intervention. Two trained researchers conducted face-to-face sessions. The questionnaires were conducted in the participants’ preferred language, either English or Chinese (in the participant’s preferred dialect). Each session lasted 45-60 minutes. Each participant was given a cash reimbursement after completing questionnaires and providing blood samples. A maximum of 9 mL of blood in ethylenediaminetetraacetic acid (EDTA) blood tubes was collected from every participant at each time point. The responses were recorded using the web-based e-Survey platform approved by the university. Sociodemographic and clinical data such as age, gender, ethnicity, marital status, employment status, education, housing type, morbidities, alcohol intake, smoking status, and physical activity were recorded. Clinical data such as TC, HDL, LDL/VLDL, fasting glucose, HbA_1c_, and BMI were also measured before and after the intervention.

#### Psychosocial Measures

The SCCII [41] assesses the process of self-care by individuals with a variety of chronic conditions. It consists of 30 items with 5-point Likert scales to evaluate self-help behavior, symptom management, health-seeking behavior, and self-care confidence. The HAI [42] focuses on how healthy and active lifestyle among the elderly is considered. The HAI includes nine components: Being self-sufficient and Living Simply, Managing Stress, Having Social Relationships and Support, Making Merit and Good Deeds, Practicing Self-care and Self-awareness, Staying Physically Active, Staying Cognitively Active, Having Social Participation, and Accepting Aging. HAI has 35 items on a 5-point scale. A higher score represents greater healthy aging levels. The HLS-SF 12 measures the competency of an individual when dealing with health-related information [43]. It consists of three domains including health care, disease prevention, and health promotion. The PES [44] is a 15-item scale developed to assess empowerment and the patient’s sense of control over their illness experience [45]. The SSQ6 measures the number of people providing support to an individual and the satisfaction level of the individual who received the support [46].

#### Brief Cognitive Tests

The MoCA is a screening instrument to detect mild cognitive impairment [47]. A study has shown that MoCA may be relatively more sensitive in detecting characteristic cognitive deficits due to cardiovascular diseases prevalent in Asian elderly, and it takes approximately 12 minutes to complete [48]. The SDMT is a sensitive processing speed test and is added to supplement MoCA for optimal cognitive screening. The SDMT is widely used and takes approximately 5 minutes to complete [49]. Cognitive ability affects the self-care behavior of patients with chronic disease. Assessment of cognitive function through the MoCA and the SDMT may help inform interventions to improve the self-care behavior in these patients.

#### Bioassay Procedures

Upon collection of blood samples, the EDTA tubes were centrifuged at 1500 *g* for 5 minutes at room temperature to separate plasma from other blood components. After centrifugation, plasma was collected, aliquoted in different tubes, and stored at –80°C for downstream analyses. Plasma glucose level was measured using the glucose assay kit (Sigma Aldrich, MK286) whereas TC, HDL, and LDL/VLDL levels were measured using the AF HDL and LDL/VLDL assay kit (Sigma Aldrich, MK331) in accordance with the manufacturer’s protocol. Absorbance was measured at 570 nm using the SpectraMax M2 microplate reader (Molecular Probes). Plasma glucose level and TC, HDL, and LDL/VLDL levels were calculated from the standard. HbA_1c_ levels were determined using a Beckman UniCel DxC600 Chemistry Analyzer (Beckman Coulter), with hemoglobin levels measured using a colorimetric method at 410 nm, and glycation levels measured using a turbidimetric immunoinhibition method at 340 nm.

### Data Analysis

Descriptive statistics, including mean (SD) and percentages, were used to summarize the demographic information and outcomes at baseline and post intervention. A paired samples *t* test was used to examine the difference between the baseline and postintervention periods and to compare outcome measures before and after implementation among participants. All analyses were conducted using RStudio (version 1.1) implementing R (version 3.4) [50], and the significance level was set at 5%.

### Ethical Issues

Ethical approval was obtained from the university’s institutional review board (H-20-028) and the hospital’s Centralised Institutional Review Board (Ref 2020/2051). Researchers explained the purpose of the study to potential participants. Informed consent was obtained from the participant prior to data collection. The participants were reassured that participation in the study was voluntary, and withdrawal from the study would not result in any negative consequences. Confidentiality and anonymity were maintained as no identifiers were recorded in the questionnaires.

## Results

### Outline of the Community-Based eHealth Program

**Figure 2:**
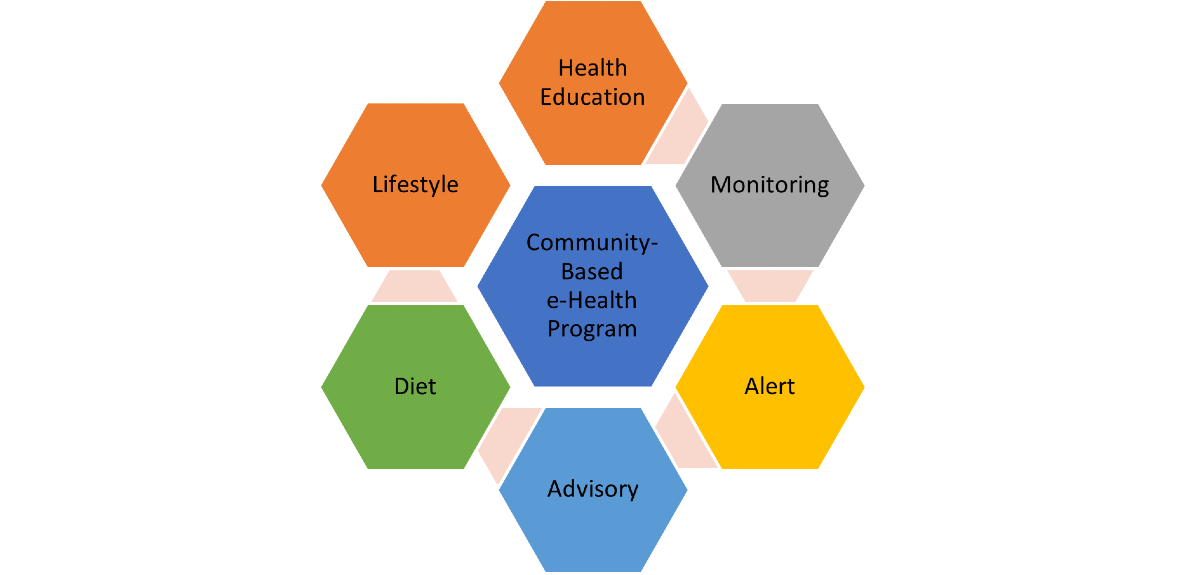
Conceptual outline of the Community-Based e-Health Program.

**Figure 3:**
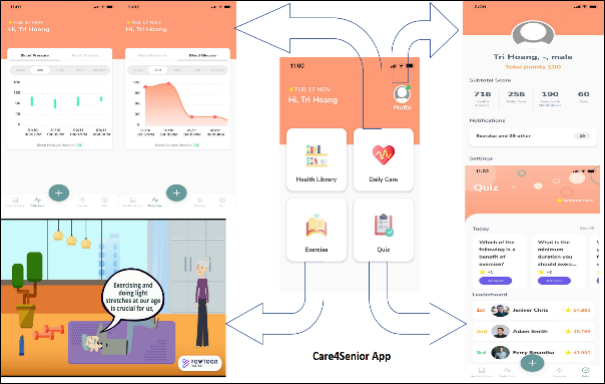
Care4Senior App - Main Screen.

**Figure 4:**
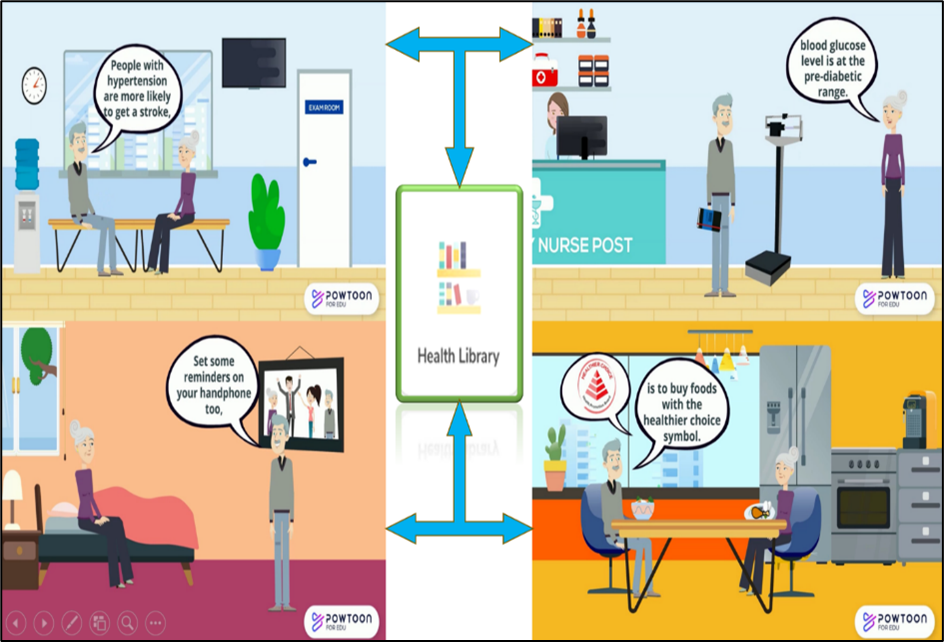
Care4Senior App - Health Library.

The CeHP consists of health education, monitoring function, and an alert and advisory system for older adults to manage their chronic conditions (Figure 2). The CeHP is an 8-week intensive program, consisting of face-to-face and eHealth (*Care4Senior* App) sessions. Face-to-face session covers health education topics such as diet, exercise, and brain health, which are available in *Care4Senior* App. *Care4Senior* App can be installed on smart devices. *Care4Senior* has unique features including a health library, daily care, exercises, quizzes, interactive videos, and administrative platform (Figure 3). *Care4Senior* consists of health education topics focusing on management of hypertension, hyperlipidemia, and diabetes, brain health, healthy diet, lifestyle modification, medication adherence, exercise, and mindfulness practice (Figure 4). Each module consists of animated videos of conversations between a fictional elderly couple, and health education topics. A prototype of *Care4Senior* App has been developed by the technical team.

#### Mode of Delivery

During the intervention, the research team conducted weekly face-to-face training and evaluated the participant’s competency in using the *Care4Senior* App. The older adults could then continue with the *Care4Senior* App at home. Researchers monitored participants’ App usage closely via the administrative platform of the App; for example, blood pressure and glucose entry, and quiz status. A reminder was sent to the participants through the phone if their blood pressure and glucose reading were not entered or were beyond the normal range. The CeHP provides a platform to improve the overall clinical outcomes for older adults living with chronic diseases by empowering them with self-care skills.

### Results of the Content Validity Test

The content validity index (CVI) was calculated, and only when both item-CVI and scale-CVI values were above 0.8, the content of the program would then be considered valid [51,52]. Our results indicate that the content of CeHP is valid since item-CVI ranged 0.86-1 and the scale-CVI was 1. Positive feedback has been received from 7 content experts that the CeHP is well-structured and covers common chronic diseases, which are helpful for older adults to gain knowledge about self-management through medication compliance and lifestyle modification. Nevertheless, content refinement was carried out for those items rated below 2. The item-CVI for revised content became 1 after reassessment by content experts. Based on the feedback, the research team fine-tuned the contents to ensure accuracy. Meanwhile, there were also concerns that the content might be overwhelming for the participants. Content experts suggested rephrasing certain terminologies for participants with lower literacy levels. Researchers readjusted the font size, reduced wordy contents, and added more pictures to be more senior-friendly.

### Results From the Pilot Evaluation

**Figure 5:**
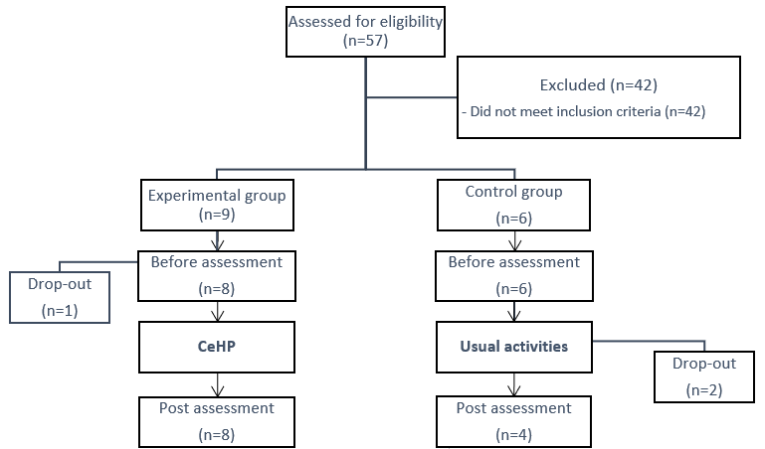
Flowchart of participant recruitment for the Community-Based e-Health Program (CeHP).

Among all screened and invited participants, a total of 15 participants enrolled in the pilot study. However, owing to drop-outs, 8 participants in the intervention (CeHP) group and 4 in the control group completed both baseline and postintervention assessments. Figure 5 shows the recruitment and program flow. Table 1 shows demographic and clinical characteristics of the participants. The Student *t* test and Pearson chi-square test revealed no significant difference between CeHP and control group participants. The mean age of the participants was 74.4 years (CeHP group) 69.75 years (control group). All participants in the CeHP group were of Chinese ethnicity, and 7 of them (88%) were female. One participant in the control group is of Malay ethnicity, and 50% of the participants were female. All participants stayed in public housing and were independent and ambulating. All participants had at least one of the chronic illnesses (hypertension, hyperlipidemia, or T2DM). Participants in the CeHP group were mostly compliant with the seminar regimes except when they had other commitments such as medical visits or work (attendance rates are shown in Table S1 in Multimedia Appendix 1). Participants also rated the design and user-friendliness of the app as above average on a 5-point scale (Table S2 in Multimedia Appendix 1).

**Table 1:** Demographic and clinical characteristics of participants in pilot evaluation.

Variables										Community-Based e-health Program group (n=8)		Control group (n=4)		*P* value^a^	
Age (years), mean (SD)										74.4 (6.22)		69.75 (8.34)		.38	
**Gender, n (%)**															.48
						Male			1 (12.5)		2 (50)				
						Female			7 (87.5)		2 (50)				
**Race, n (%)**															.71
					Chinese				8 (100)		3 (75)				
					Malay				0		1 (25)				
**Marital status, n (%)**															.22
								Single, separated, or divorced	3 (37.5)		0				
								Married	1 (12.5)		2 (50)				
								Widowed	4 (50)		2 (50)				
**Highest education level, n (%)**															>.99
							None or primary education		6 (75)		3 (75)				
							Secondary school and above		2 (25)		1 (25)				
**Employment status, n (%)**															>.99
				Working					1 (12.5)		0				
				Not working or retired					7 (87.5)		4 (100)				
**Housing type, n (%)**															.15
			HDB^b^ studio apartment						3 (37.5)		4 (100)				
			HDB 3-room apartment and above						5 (62.5)		0				
**Living status, n (%)**															.28
		Alone							4 (50)		0				
		With others							4 (50)		4 (100)				
**Physical exercise, n (%)**															.71
	>3 times per week								8 (100)		3 (75)				
	Never								0		1 (25)				
Current smoker, n (%)										0 (0)		1 (25)		.71	
Regular drinker, n (%)										0 (0)		1 (25)		.71	
Hypertension, n (%)										6 (75)		4 (100)		.78	
Hyperlipidemia, n (%)										7 (87.5)		3 (75)		>.99	
Type 2 diabetes, n (%)										3 (37.5)		4 (100)		.15	

^a^Age was compared using the Student *t* test, whereas other categorical characteristics were compared using the Pearson chi-square test or the Fisher exact test.

^b^HDB: Housing and Development Board.

Table 2 shows the psychosocial, cognitive, and blood test findings before and after the intervention. Participants in the CeHP group demonstrated improvements in fasting glucose, HbA_1c_, TC, LDL/VLDL, BMI, 3 of 4 SCCII indices (in the following domains: Maintenance, Monitoring, and Management), and HAI and PES scores, though the changes are not significant. Among control group participants, the scores for the two domains (Management and Confidence) from SCCII and HLS-SF 12 decreased after the intervention. The participants’ (control group) fasting glucose and TC levels were also higher post intervention than during baseline assessment; however, these differences were not significant.

**Table 2:** Mean scores of study outcomes.

Measures			Community-Based e-Health Program group (n=8), mean (SD)			Control group (n=4), mean (SD)		
			Baseline	Post intervention	*P* value^a^	Baseline	Post intervention	*P* value^a^
**Psychosocial measures**								
	**Self-care of Chronic Illness Inventory indices**							
		Maintenance	85.94 (10.83)	93.36 (4.85)	.09	83.59 (4.69)	87.5 (14.66)	.56
		Monitoring	67.29 (24.53)	70.0 (13.09)	.81	52.92 (13.77)	63.5 (16.56)	.28
		Management	54.0 (10.47)	61 (8.21)	.13	60 (10.83)	54 (12.44)	.18
		Confidence	87.81 (9.95)	87.5 (10.69)	.95	83.13 (10.08)	78.75 (12.67)	.37
	Healthy Aging Instrument		147.63 (17.07)	149.75 (6.82)	.68	136.25 (23.26)	148.25 (15.59)	.10
	Patient Empowerment Scale		40.63 (3.38)	43.75 (8.31)	.29	39.50 (2.08)	47.25 (7.54)	.08
	Social Support Questionnaire, 6 items satisfaction total score		29.63 (4.44)	28.75 (4.56)	.61	27.75 (5.68)	28.25 (4.79)	.70
	Health Literacy Survey Short Form (HLS-SF12) Index		30.56 (3.32)	30.38 (4.03)	.78	29.51 (6.35)	26.39 (6.0)	.06
	**Cognitive tests**							
		Montreal cognitive assessment total score	25.13 (3.40)	23.13 (4.36)	.20	19.75 (3.30)	20.0 (5.72)	.87
		Symbol digit modalities test score	27.5 (9.68)	26.0 (14.25)	.56	18.75 (2.99)	19.75 (3.59)	.51
BMI			25.19 (3.76)	24.78 (4.06)	.35	25.36 (3.48)	.20	.68
**Biomarkers**								
	Fasting glucose (mg/dL)		90.77 (28.32)	84.12 (13.47)	.26	110.54 (24.95)	124.88 (71.36)	.58
	Glycated hemoglobin (%)		6.49 (0.84)	6.31 (0.60)	.11	8.13 (0.49)	7.18 (0.63)	.07
	Total cholesterol (mg/dL)		115.30 (7.76)	114.99 (4.76)	.94	110.29 (8.12)	111.53 (9.14)	.68
	High-density lipoprotein cholesterol (mg/dL)		35.36 (10.46)	31.38 (7.41)	.29	31.20 (5.83)	32.21 (4.52)	.80
	Low-density lipoprotein/very-low-density lipoprotein cholesterol (mg/dL)		103.91 (10.49)	99.10 (16.24)	.25	93.69 (12.55)	88.86 (17.35)	.28

^a^*P* values determined through the Student paired *t* test.

## Discussion

### Principal Findings

This paper illustrates a systematic 3-step process of developing a community-based health education program coupled with the use of a smart-device application. Development of the intervention consists of a theoretical framework, a client-centric participatory action research process, and psychometric testing. The rigorous process ensured the validity of the intervention, and explicitly reporting the detailed description of the intervention could facilitate replication of the intervention in the future.

The prevalence of chronic diseases is increasing among the older population. Hypertension, hyperlipidemia, and T2DM are the most common chronic conditions among community-dwelling older adults. The progression of diseases and impact on quality of life can be tapered off by active treatment and self-management. By promoting health literacy and awareness of community health resources, it is feasible to reduce debilitating complications of poorly controlled chronic conditions and subsequent hospitalization, which contributes to the burden of the health care system [53].

The results from the pilot test revealed that the CeHP was feasible and potentially effective in improving self-management capabilities of older adults. The pilot test demonstrated improvements in fasting glucose, HbA_1c_, TC, LDL/VLDL, BMI, SCCII indices, HAI scores, although these changes were not significant, which could be due to a small sample size. eHealth interventions have gained popularity among older adults in the recent years. Research has shown that daily monitoring via eHealth interventions increased older adults’ confidence, control, awareness in managing their conditions, prompted more communication with their doctors, and using monitoring records to review their medications [53,54]. Hence, participants were more proactive in managing their conditions.

The results of the pilot test showed improvements in the PES score, albeit not significant. Research has shown that eHealth interventions improved older adults’ self-efficacy for health-decision making and patient-provider communication [55]. As a result, older adults are empowered to take charge in managing their chronic conditions. eHealth interventions have shown improvement in chronic disease self-management and reduced health care utilization [56], which evidenced that eHealth interventions are feasible to be implemented among community-dwelling older adults and are beneficial in reducing health care costs.

With the high attendance rate (86% in average), high overall satisfaction toward the App (75%), and positive user feedback (Multimedia Appendix 1), the pilot test provided evidence that CeHP has excellent features as a senior-friendly App to deliver health-related information to the older adults. With the rapid adoption of information technology in health care, more technology-based interventions will be utilized in the delivery of care. Older adults are a large consumer group for health care services. Hence, service providers need to consider various aspects to facilitate uptake, such as user-friendly e-interventions for older adults and appropriate user training [55].

It is noteworthy that many older adults are not technologically savvy despite the rapid increase in internet-based users among the older adult population [55]. Hence, older adults may require training and support initially in using eHealth interventions [57], as observed by our researchers during the pilot study. Research has shown that older adults are more confident in maneuvering the internet after undergoing the training [56,58]. Portz and LaMendola [57] reported that the average duration of web-based participation among older adults was longer than that in younger cohorts, which could be an indication that older adults tend to be committed to eHealth programs to improve their health outcomes. Multiple studies have shown that older adults living alone reported poor health, which could predict increased hospital utilization [56]. By attending face-to-face group seminar sessions, participants may gain social support from fellow groupmates, which may serve as a complementary strategy for enhancing individual’s ability of self-management through social network to better manage chronic diseases [15,16,18,19].

A systematic review reported that eHealth programs provide support and feedback for a healthy lifestyle and highlighted the evidence on the facilitating factors and barriers [59]. The barriers are lack of motivation and support; however, strong motivation, adequate support, and feedback are facilitating factors for the continuity of eHealth programs [59]. The most frequent motivator is feedback from professionals or peers on the extent to which people have achieved their goals. Hence, eHealth interventions can tap on the resources from volunteers in the community to provide support to the participants. Prior training is necessary to equip the peer volunteers with essential skills [53].

The larger-scale intervention after this pilot evaluation will be compared against a control group in a randomized controlled trial. Owing to low education level in older adults (75% with primary school of below in the pilot trial), we anticipate barriers for these older adults to use technological devices. This will be countered by having face-to-face sessions to teach the older adults in using the *Care4Senior* App. In addition, the app was also developed in both English and Chinese (Mandarin) languages to cater to the needs of elderly population in Singapore.

### Limitations

As a pilot evaluation, this phase of the study was carried out to assess its feasibility and refine its structure and operations. The results of the pilot test may be biased owing to the small sample size and the predisposition of the participants being already health conscious. The 8-week duration may also be too short to elicit significant changes in health behaviors that improve health outcomes.

### Conclusions

A large proportion of older adults are living with multiple chronic diseases, and thus managing their health in the community is a major public health concern. The CeHP engaged and empowered older adults living in the community to manage their chronic conditions. The rigorous process of program development and pilot evaluation provided valid evidence to extend CeHP to a subsequent larger-scale trial to encourage self-management, reduce debilitating complications of poorly controlled chronic diseases, promote healthy longevity and social support, and reduce health care costs. In the future, eHealth interventions can tap on the resources from volunteers in the community to provide support to the older adults.

## Appendix

Multimedia Appendix 1 Face-to-face seminar attendance rates and participants’ ratings regarding app design and user-friendliness.

## Abbreviations

CeHP: Community-based e-Health Program
EDTA: ethylenediaminetetraacetic acid
HAI: Healthy Aging Instrument
HbA_1c_: glycated hemoglobin
HDL: high-density lipoprotein
HLS-SF 12: Short-Form Health Literacy Scale, 12 Items
LDL: low-density lipoprotein
MoCA: Montreal Cognitive Assessment
PES: Patient Empowerment Scale
SAC: senior activity center
SCCII: Self-care of Chronic Illness Inventory
SDMT: Symbol Digit Modalities Test
SMP: self-management program
SSQ6: Social Support Questionnaire, 6 items
T2DM: type 2 diabetes mellitus
TC: total cholesterol
VLDL: very-low-density lipoprotein
