# Supplementary material for: Development of a Community-Based e-Health Program for Older Adults With Chronic Diseases: Pilot Pre-Post Study
Source: JMIR Aging. 2022 Jan 17;5(1):e33118. doi: 10.2196/33118 (PMC8804958; doi:10.2196/33118)
Supplement: Multimedia Appendix 1 [file aging_v5i1e33118_app1.docx]

Supplementary Table S1: Face-to-face seminar attendance rates.

| **Participant ID** | **Attendance - out of 8 sessions, (%)** | **Reason** |
| --- | --- | --- |
| P21 | 7 / 8 (87.5%) | Work commitment |
| P23 | 8 / 8 (100%) | FULL ATTENDANCE |
| P24 | 5 / 8 (62.5%) | Medical visits and procedures |
| P25 | 7 / 8 (87.5%) | Medical visit |
| P26 | 7 / 8 (87.5%) | Medical visit |
| P27 | 7 / 8 (87.5%) | Feeling unwell |
| P28 | 7 / 8 (87.5%) | Feeling unwell |
| P29 | 7 / 8 (87.5%) | Feeling unwell |

Supplementary Table S2: Participants’ ratings regarding App design and user-friendliness.

| **Aspect** | **Average rating from 6 users** |
| --- | --- |
| Overall satisfaction towards the App | 7.5 / 10 |
| Overall ease of use | All rated “Neither easy nor difficult” |
| System speed | 2.5 / 5 |
| Ease of navigation | 3.5 / 5 |
| Quality of information | 3.5 / 5 |
| App design | 4.3 / 5 |
